# Supplementary material for: Do the Shuffle: Expanding the Synthetic Biology Toolkit for Shufflon-like Recombination Systems
Source: ACS Synth Biol. 2025 Jan 27;14(2):363–72. doi: 10.1021/acssynbio.4c00790 (PMC11852207; doi:10.1021/acssynbio.4c00790)
Supplement: Supplementary file 3 — sb4c00790_si_003.pdf [file sb4c00790_si_003.pdf]

Supporting Information

Manuscript Title:
Do the shuffle: Expanding the synthetic biology toolkit for shufflon-like recombination systems

Author List
Jan Katalinić[1], Morgan Richards[1], Alex Auyang[1], James H. Millett[1], Manjunatha Kogenaru[1], Nikolai Windbichler[1,*]

*nikolai.windbichler@imperial.ac.uk

Affiliations
1. Department of Life Sciences, Imperial College London, SW7 2AZ, UK

Figure S1

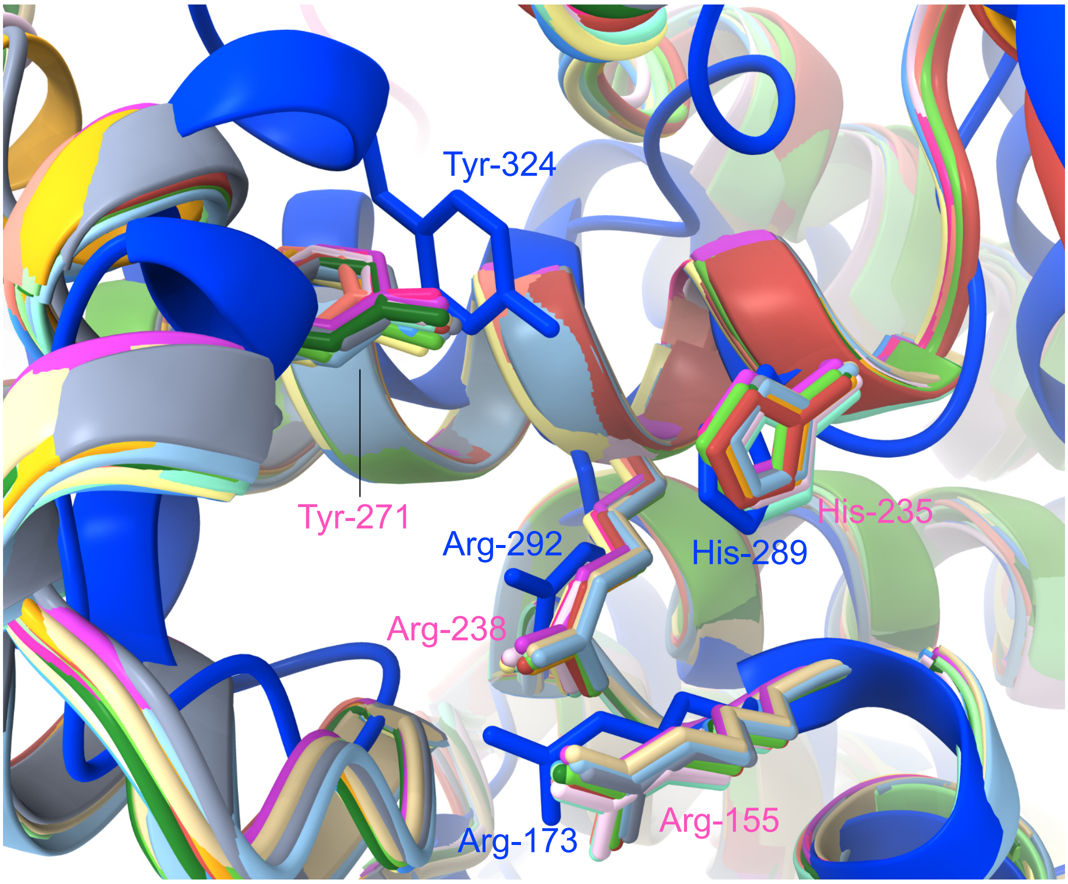

■: Cre ■: Rci else: homologs

Figure S2

A

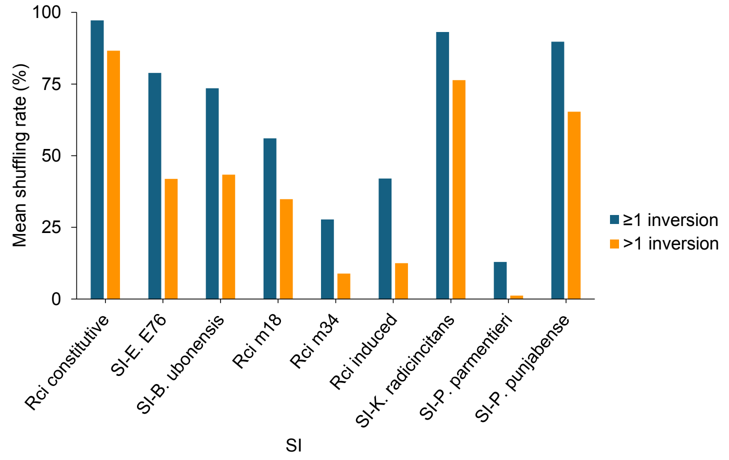

100
75
50
25
0
Mean shuffling rate (%)
≥1 inversion
>1 inversion
Rci constitutive
SI-E. E76
SI-B. ubonensis
Rci m18
Rci m34
Rci induced
SI-K. radicincitans
SI-P. parmentieri
SI-P . punjabense
SI

B

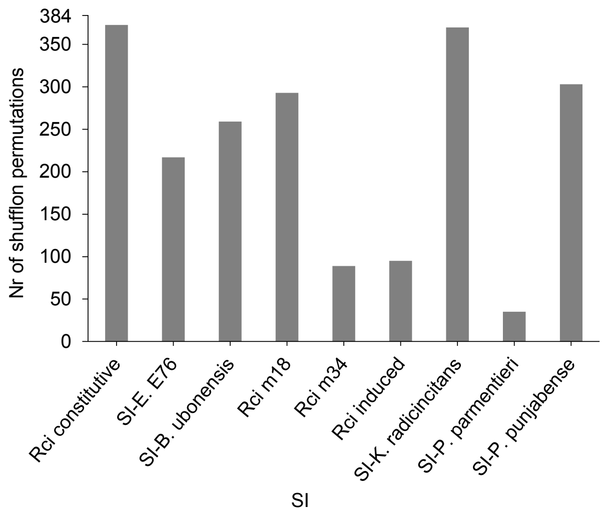

384
350
300
250
200
150
100
50
0
Nr of shufflon permutations
Rci constitutive
SI-E. E76
SI-B. ubonensis
Rci m18
Rci m34
Rci induced
SI-K. radicincitans
SI-P. parmentieri
SI-P . punjabense
SI

Figure S3

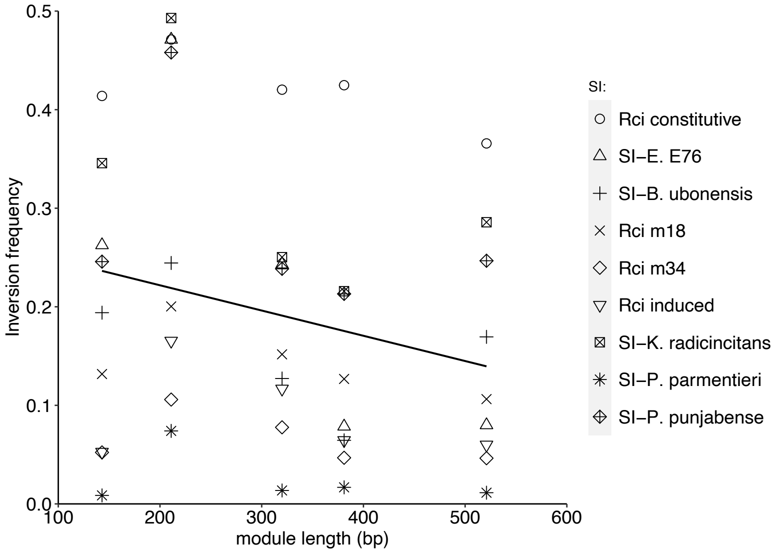

0.5
0.4
0.3
0.2
0.1
0.0
Inversion frequency
100
200
300
400
500
600
module length (bp)
SI:
Rci constitutive
SI−E. E76
SI−B. ubonensis
Rci m18
Rci m34
Rci induced
SI−K. radicincitans
SI−P. parmentieri
SI−P. punjabense

Supplementary Figure 1. Conservation of catalytic residues in Rci homologs. Catalytic tetrad Arg-His-Arg-Tyr displayed in stick style in an overlay of protein models of *Rci*, *Rci* homologs, and the protein structure of *Cre*.

Supplementary Figure 2. Analysis of shuffled libraries. A. For each SI, the mean shuffling rate (orange) which measures the proportion of analysed library members which contain shufflons that are not in the original (unshuffled) configuration as well as the multiple-shuffling rate (blue) which measures the proportion library members that are the product of more than 1 inversion are shown for the pooled data of both biological replicates. **B.** Permutations were quantified from 4000 randomly sampled library members for each SI from the pooled data of both biological replicates. There are a maximum of 384 possible permutations for a five-module shufflon.

Supplementary Figure 3. Analysis of single module inversions. For each SI, those library members from both pooled biological replicates that contained the reference module configuration (in either inverted or non-inverted form) were extracted and then randomly downsampled to the smallest library of 2201 members. A multiple regression analysis indicated that module length ($p<0.00361$) and SI ($p<1.484\times10^{-9}$) were regression coefficients significantly explaining the module inversion frequencies.

Table S1: Reporter and inducible expression vector assembly primers

Primer ID	Sequence (5' to 3')
Universal FWD2	CAGGAGCGCACCATCTTC
Universal FWD3	GAGATGGCCGATACTGTG
Universal FWD4	CGACGAGGACTTCAGCTC
Universal FWD5	CCTACGAGACATTCAAGAGC
Rci FWD1	CTAGTGGATCCAGTGTGGTGGAATTCTTCTCTGCAAGCGTGCCAATCCGGTACGTGGGGTACCGACGTAAACGGCCACAAG
Rci REV1	CTTGAAGAAGATGGTGCGCTCCTGGGTACCTTCTCTGCAAGCGTGCCAATCCGGTACGTGGTAGGTCAGGGTGGTCACG
Rci REV2	GGGAATCACAGTATCGGCCATCTCGGTACCCCACGTACCGGATTGGCACGCTTGCAGAGAATGATGCCGTTCTTCTGCTTG
Rci REV3	GCGATAGAGCTGAAGTCCTCGTCGGGTACCTTCTCTGCAAGCGTGCCAATCCGGTACGTGGCCAGGATTCTCCTCGACG
Rci REV4	TGATGCTCTTGAATGTCTCGTAGGGGTACCCCACGTACCGGATTGGCACGCTTGCAGAGAAGCTTTGACAGCCTGGCTC
Rci REV5	CCAGTGACTACATCTAGACTCGAGTTTCTCTGCAAGCGTGCCAATCCGGTACGTGGCTGCTGGAACTCGCTGTTG
H. paralvei FWD1	CTAGTGGATCCAGTGTGGTGGAATTCTTTTGTCAATCCTGTCAATCCGGCGTGTGGGGTACCGACGTAAACGGCCACAAG
H. paralvei REV1	CTTGAAGAAGATGGTGCGCTCCTGGGTACCTTTTGTCAATCCTGTCAATCCGGCGTGTGGTAGGTCAGGGTGGTCACG
H. paralvei REV2	GGGAATCACAGTATCGGCCATCTCGGTACCCCACACGCCGGATTGACAGGATTGACAAAATGATGCCGTTCTTCTGCTTG
H. paralvei REV3	GCGATAGAGCTGAAGTCCTCGTCGGGTACCTTTTGTCAATCCTGTCAATCCGGCGTGTGGCCAGGATTCTCCTCGACG
H. paralvei REV4	TGATGCTCTTGAATGTCTCGTAGGGGTACCCCACACGCCGGATTGACAGGATTGACAAAAGCTTTGACAGCCTGGCTC
H. paralvei REV5	CCAGTGACTACATCTAGACTCGAGTTTTTGTCAATCCTGTCAATCCGGCGTGTGGCTGCTGGAACTCGCTGTTG
Y. pseudo. FWD1	CTAGTGGATCCAGTGTGGTGGAATTCATTTGTCAATCTTGTCAATCCGGTGTGTGGGGTACCGACGTAAACGGCCACAAG
Y. pseudo. REV1	CTTGAAGAAGATGGTGCGCTCCTGGGTACCATTTGTCAATCTTGTCAATCCGGTGTGTGGTAGGTCAGGGTGGTCACG
Y. pseudo. REV2	GGGAATCACAGTATCGGCCATCTCGGTACCCCACACACCGGATTGACAAGATTGACAAATTGATGCCGTTCTTCTGCTTG
Y. pseudo. REV3	GCGATAGAGCTGAAGTCCTCGTCGGGTACCATTTGTCAATCTTGTCAATCCGGTGTGTGGCCAGGATTCTCCTCGACG
Y. pseudo. REV4	TGATGCTCTTGAATGTCTCGTAGGGGTACCCCACACACCGGATTGACAAGATTGACAAATGCTTTGACAGCCTGGCTC
Y. pseudo. REV5	CCAGTGACTACATCTAGACTCGAGTATTTGTCAATCTTGTCAATCCGGTGTGTGGCTGCTGGAACTCGCTGTTG
E. clo/hor FWD1*	CTAGTGGATCCAGTGTGGTGGAATTCGACAAAGGCGCGACACTTTCTTGTCAATCCGGTGTGTGGGGTACCGACGTAAACGGCCACAAG
E. clo/hor REV1*	CTTGAAGAAGATGGTGCGCTCCTGGGTACCGACAAAGGCGCGACACTTTCTTGTCAATCCGGTGTGTGGTAGGTCAGGGTGGTCACG
E. clo/hor REV2*	GGGAATCACAGTATCGGCCATCTCGGTACCCCACACACCGGATTGACAAGAAAGTGTCGCGCCTTTGTCTGATGCCGTTCTTCTGCTTG
E. clo/hor REV3*	GCGATAGAGCTGAAGTCCTCGTCGGGTACCGACAAAGGCGCGACACTTTCTTGTCAATCCGGTGTGTGGCCAGGATTCTCCTCGACG
E. clo/hor REV4*	TGATGCTCTTGAATGTCTCGTAGGGGTACCCCACACACCGGATTGACAAGAAAGTGTCGCGCCTTTGTCGCTTTGACAGCCTGGCTC
E. clo/hor REV5*	CCAGTGACTACATCTAGACTCGAGTGACAAAGGCGCGACACTTTCTTGTCAATCCGGTGTGTGGCTGCTGGAACTCGCTGTTG
E. E76 FWD1	CTAGTGGATCCAGTGTGGTGGAATTCGCATTAGCAATTAAATAAACCTGTCAATCCGGTGTGTGGGGTACCGACGTAAACGGCCACAAG
E. E76 REV1	CTTGAAGAAGATGGTGCGCTCCTGGGTACCGCATTAGCAATTAAATAAACCTGTCAATCCGGTGTGTGGTAGGTCAGGGTGGTCACG
E. E76 REV2	GGGAATCACAGTATCGGCCATCTCGGTACCCCACACACCGGATTGACAGGTTTATTTAATTGCTAATGCTGATGCCGTTCTTCTGCTTG
E. E76 REV3	GCGATAGAGCTGAAGTCCTCGTCGGGTACCGCATTAGCAATTAAATAAACCTGTCAATCCGGTGTGTGGCCAGGATTCTCCTCGACG
E. E76 REV4	TGATGCTCTTGAATGTCTCGTAGGGGTACCCCACACACCGGATTGACAGGTTTATTTAATTGCTAATGCGCTTTGACAGCCTGGCTC
E. E76 REV5	CCAGTGACTACATCTAGACTCGAGTGCATTAGCAATTAAATAAACCTGTCAATCCGGTGTGTGGCTGCTGGAACTCGCTGTTG
M. glu. FWD1	CTAGTGGATCCAGTGTGGTGGAATTCTTTTGTCAATCCTGTCAATCCGGCGTGTGGGGTACCGACGTAAACGGCCACAAG
M. glu. REV1	CTTGAAGAAGATGGTGCGCTCCTGGGTACCTTTTGTCAATCCTGTCAATCCGGCGTGTGGTAGGTCAGGGTGGTCACG
M. glu. REV2	GGGAATCACAGTATCGGCCATCTCGGTACCCCACACGCCGGATTGACAGGATTGACAAAATGATGCCGTTCTTCTGCTTG
M. glu. REV3	GCGATAGAGCTGAAGTCCTCGTCGGGTACCTTTTGTCAATCCTGTCAATCCGGCGTGTGGCCAGGATTCTCCTCGACG
M. glu. REV4	TGATGCTCTTGAATGTCTCGTAGGGGTACCCCACACGCCGGATTGACAGGATTGACAAAAGCTTTGACAGCCTGGCTC
M. glu. REV5	CCAGTGACTACATCTAGACTCGAGTTTTTGTCAATCCTGTCAATCCGGCGTGTGGCTGCTGGAACTCGCTGTTG
B. ubo. FWD1	CTAGTGGATCCAGTGTGGTGGAATTCTTTTGTCAATCCTGTCAATCCGGCGTGTGGGGTACCGACGTAAACGGCCACAAG
B. ubo. REV1	CTTGAAGAAGATGGTGCGCTCCTGGGTACCTTTTGTCAATCCTGTCAATCCGGCGTGTGGTAGGTCAGGGTGGTCACG
B. ubo. REV2	GGGAATCACAGTATCGGCCATCTCGGTACCCCACACGCCGGATTGACAGGATTGACAAAATGATGCCGTTCTTCTGCTTG
B. ubo. REV3	GCGATAGAGCTGAAGTCCTCGTCGGGTACCTTTTGTCAATCCTGTCAATCCGGCGTGTGGCCAGGATTCTCCTCGACG
B. ubo. REV4	TGATGCTCTTGAATGTCTCGTAGGGGTACCCCACACGCCGGATTGACAGGATTGACAAAAGCTTTGACAGCCTGGCTC
B. ubo. REV5	CCAGTGACTACATCTAGACTCGAGTTTTTGTCAATCCTGTCAATCCGGCGTGTGGCTGCTGGAACTCGCTGTTG
P. parvum FWD1	CTAGTGGATCCAGTGTGGTGGAATTCTTTTGTCAATCCTGTCAATCCGGCGTGTGGGGTACCGACGTAAACGGCCACAAG
P. parvum REV1	CTTGAAGAAGATGGTGCGCTCCTGGGTACCTTTTGTCAATCCTGTCAATCCGGCGTGTGGTAGGTCAGGGTGGTCACG
P. parvum REV2	GGGAATCACAGTATCGGCCATCTCGGTACCCCACACGCCGGATTGACAGGATTGACAAAATGATGCCGTTCTTCTGCTTG
P. parvum REV3	GCGATAGAGCTGAAGTCCTCGTCGGGTACCTTTTGTCAATCCTGTCAATCCGGCGTGTGGCCAGGATTCTCCTCGACG
P. parvum REV4	TGATGCTCTTGAATGTCTCGTAGGGGTACCCCACACGCCGGATTGACAGGATTGACAAAAGCTTTGACAGCCTGGCTC
P. parvum REV5	CCAGTGACTACATCTAGACTCGAGTTTTTGTCAATCCTGTCAATCCGGCGTGTGGCTGCTGGAACTCGCTGTTG
P. cit. FWD1	CTAGTGGATCCAGTGTGGTGGAATTCTTTTGTCAATCCTGTCAATCCGGCGTGTGGGGTACCGACGTAAACGGCCACAAG
P. cit. REV1	CTTGAAGAAGATGGTGCGCTCCTGGGTACCTTTTGTCAATCCTGTCAATCCGGCGTGTGGTAGGTCAGGGTGGTCACG
P. cit. REV2	GGGAATCACAGTATCGGCCATCTCGGTACCCCACACGCCGGATTGACAGGATTGACAAAATGATGCCGTTCTTCTGCTTG
P. cit. REV3	GCGATAGAGCTGAAGTCCTCGTCGGGTACCTTTTGTCAATCCTGTCAATCCGGCGTGTGGCCAGGATTCTCCTCGACG
P. cit. REV4	TGATGCTCTTGAATGTCTCGTAGGGGTACCCCACACGCCGGATTGACAGGATTGACAAAAGCTTTGACAGCCTGGCTC
P. cit. REV5	CCAGTGACTACATCTAGACTCGAGTTTTTGTCAATCCTGTCAATCCGGCGTGTGGCTGCTGGAACTCGCTGTTG
P. kor. FWD1	CTAGTGGATCCAGTGTGGTGGAATTCTTTTGTCAATCCTGTCAATCCGGCGTGTGGGGTACCGACGTAAACGGCCACAAG
P. kor. REV1	CTTGAAGAAGATGGTGCGCTCCTGGGTACCTTTTGTCAATCCTGTCAATCCGGCGTGTGGTAGGTCAGGGTGGTCACG
P. kor. REV2	GGGAATCACAGTATCGGCCATCTCGGTACCCCACACGCCGGATTGACAGGATTGACAAAATGATGCCGTTCTTCTGCTTG
P. kor. REV3	GCGATAGAGCTGAAGTCCTCGTCGGGTACCTTTTGTCAATCCTGTCAATCCGGCGTGTGGCCAGGATTCTCCTCGACG
P. kor. REV4	TGATGCTCTTGAATGTCTCGTAGGGGTACCCCACACGCCGGATTGACAGGATTGACAAAAGCTTTGACAGCCTGGCTC
P. kor. REV5	CCAGTGACTACATCTAGACTCGAGTTTTTGTCAATCCTGTCAATCCGGCGTGTGGCTGCTGGAACTCGCTGTTG
P. aer. FWD1	CTAGTGGATCCAGTGTGGTGGAATTCTTTTGTCAATCCTGTCAATCCGGCGTGTGGGGTACCGACGTAAACGGCCACAAG
P. aer. REV1	CTTGAAGAAGATGGTGCGCTCCTGGGTACCTTTTGTCAATCCTGTCAATCCGGCGTGTGGTAGGTCAGGGTGGTCACG
P. aer. REV2	GGGAATCACAGTATCGGCCATCTCGGTACCCCACACGCCGGATTGACAGGATTGACAAAATGATGCCGTTCTTCTGCTTG
P. aer. REV3	GCGATAGAGCTGAAGTCCTCGTCGGGTACCTTTTGTCAATCCTGTCAATCCGGCGTGTGGCCAGGATTCTCCTCGACG
P. aer. REV4	TGATGCTCTTGAATGTCTCGTAGGGGTACCCCACACGCCGGATTGACAGGATTGACAAAAGCTTTGACAGCCTGGCTC
P. aer. REV5	CCAGTGACTACATCTAGACTCGAGTTTTTGTCAATCCTGTCAATCCGGCGTGTGGCTGCTGGAACTCGCTGTTG
P. parm. FWD1	CTAGTGGATCCAGTGTGGTGGAATTCTTGAACCGCTTGTGAGCACCTTGCCAATCCGGTGCGTGGGGTACCGACGTAAACGGCCACAAGTT
P. parm. REV1	CTTGAAGAAGATGGTGCGCTCCTGGGTACCTTGAACCGCTTGTGAGCACCTTGCCAATCCGGTGCGTGGTAGGTCAGGGTGGTCACGAG
P. parm. REV2	GGGAATCACAGTATCGGCCATCTCGGTACCCCACGCACCGGATTGGCAAGGTGCTCACAAGCGGTTCAATGATGCCGTTCTTCTGCTTG
P. parm. REV3	GCGATAGAGCTGAAGTCCTCGTCGGGTACCTTGAACCGCTTGTGAGCACCTTGCCAATCCGGTGCGTGGCCAGGATTCTCCTCGACGTC
P. parm. REV4	TGATGCTCTTGAATGTCTCGTAGGGGTACCCCACGCACCGGATTGGCAAGGTGCTCACAAGCGGTTCAAGCTTTGACAGCCTGGCTCG
P. parm. REV5	CCAGTGACTACATCTAGACTCGAGTTTGAACCGCTTGTGAGCACCTTGCCAATCCGGTGCGTGGCTGCTGGAACTCGCTGTTGT
P. punj. FWD1	CTAGTGGATCCAGTGTGGTGGAATTCAATGTTGGTGCAATACTTTCTTGCCAATCCGGTGCGTGGGGTACCGACGTAAACGGCCACAAGTT
P. punj. REV1	CTTGAAGAAGATGGTGCGCTCCTGGGTACCAATGTTGGTGCAATACTTTCTTGCCAATCCGGTGCGTGGTAGGTCAGGGTGGTCACGAG
P. punj. REV2	GGGAATCACAGTATCGGCCATCTCGGTACCCCACGCACCGGATTGGCAAGAAAGTATTGCACCAACATTTGATGCCGTTCTTCTGCTTG
P. punj. REV3	GCGATAGAGCTGAAGTCCTCGTCGGGTACCAATGTTGGTGCAATACTTTCTTGCCAATCCGGTGCGTGGCCAGGATTCTCCTCGACGTC
P. punj. REV4	TGATGCTCTTGAATGTCTCGTAGGGGTACCCCACGCACCGGATTGGCAAGAAAGTATTGCACCAACATTGCTTTGACAGCCTGGCTCG
P. punj. REV5	CCAGTGACTACATCTAGACTCGAGTAATGTTGGTGCAATACTTTCTTGCCAATCCGGTGCGTGGCTGCTGGAACTCGCTGTTGT
K. rad. FWD1	CTAGTGGATCCAGTGTGGTGGAATTCGCGTCTGGCGCAATACTTTCCTGTCAATCCGGTGTGTGGGGTACCGACGTAAACGGCCACAAGTT
K. rad. REV1	CTTGAAGAAGATGGTGCGCTCCTGGGTACCGCGTCTGGCGCAATACTTTCCTGTCAATCCGGTGTGTGGTAGGTCAGGGTGGTCACGAG
K. rad. REV2	GGGAATCACAGTATCGGCCATCTCGGTACCCCACACACCGGATTGACAGGAAAGTATTGCGCCAGACGCTGATGCCGTTCTTCTGCTTG
K. rad. REV3	GCGATAGAGCTGAAGTCCTCGTCGGGTACCGCGTCTGGCGCAATACTTTCCTGTCAATCCGGTGTGTGGCCAGGATTCTCCTCGACGTC
K. rad. REV4	TGATGCTCTTGAATGTCTCGTAGGGGTACCCCACACACCGGATTGACAGGAAAGTATTGCGCCAGACGCGCTTTGACAGCCTGGCTCG
K. rad. REV5	CCAGTGACTACATCTAGACTCGAGTGCGTCTGGCGCAATACTTTCCTGTCAATCCGGTGTGTGGCTGCTGGAACTCGCTGTTGT
AraC-pBAD FWD	GCCCTTCGCTGAAGACGTTTATGACAACTTGACGGCTACA
AraC-pBAD REV	CTCTCTTCCGGTATGGAGAAACAGTAGAGAGT
Homolog FWD universal	CTCCATACCGGAAGAGAGACAATTCAGGGTG
K. rad. EXP REV	GTTTTATTTGATGCCTCTACTTAGGTACCGTTGTCGGTACC
P. parm. & P. punj. EXP REV	GTTTTATTTGATGCCTCTACTTACAGCGGAGAGATCAGCAG
Inducible Rci EXP REV	GTTTTATTTGATGCCTCTACTTACAGCGGTGTGCTG

*: SI-E. cloacae and SI-E. hormaechei have identical sfx sites.
